# Inhibitory Effect of Flavonolignans on the P2Y12 Pathway in Blood Platelets

**DOI:** 10.3390/molecules23020374

**Published:** 2018-02-10

**Authors:** Michal Bijak, Rafal Szelenberger, Angela Dziedzic, Joanna Saluk-Bijak

**Affiliations:** Department of General Biochemistry, Faculty of Biology and Environmental Protection, University of Lodz, Pomorska 141/143, 90-236 Lodz, Poland; rafal.szelenberger@unilodz.eu (R.S.); angela.dziedzic@unilodz.eu (A.D.); joanna.saluk@biol.uni.lodz.pl (J.S.-B.)

**Keywords:** blood platelets, ADP, P2Y12, silybin, silychristin, silydianin, flavonolignans

## Abstract

Adenosine diphosphate (ADP) is the major platelet agonist, which is important in the shape changes, stability, and growth of the thrombus. Platelet activation by ADP is associated with the G protein-coupled receptors P2Y1 and P2Y12. The pharmacologic blockade of the P2Y12 receptor significantly reduces the risk of peripheral artery disease, myocardial infarction, ischemic stroke, and vascular death. Recent studies demonstrated the inhibition of ADP-induced blood platelet activation by three major compounds of the flavonolignans group: silybin, silychristin, and silydianin. For this reason, the aim of the current work was to verify the effects of silybin, silychristin, and silydianin on ADP-induced physiological platelets responses, as well as mechanisms of P2Y12-dependent intracellular signal transduction. We evaluated the effect of tested flavonolignans on ADP-induced blood platelets’ aggregation in platelet-rich plasma (PRP) (using light transmission aggregometry), adhesion to fibrinogen (using the static method), and the secretion of PF-4 (using the ELISA method). Additionally, using the double labeled flow cytometry method, we estimated platelet vasodilator-stimulated phosphoprotein (VASP) phosphorylation. We demonstrated a dose-dependent reduction of blood platelets’ ability to perform ADP-induced aggregation, adhere to fibrinogen, and secrete PF-4 in samples treated with flavonolignans. Additionally, we observed that all of the tested flavonolignans were able to increase VASP phosphorylation in blood platelets samples, which is correlated with P2Y12 receptor inhibition. All of these analyses show that silychristin and silybin have the strongest inhibitory effect on blood platelet activation by ADP, while silydianin also inhibits the ADP pathway, but to a lesser extent. The results obtained in this study clearly demonstrate that silybin, silychristin, and silydianin have inhibitory properties against the P2Y12 receptor and block ADP-induced blood platelet activation.

## 1. Introduction

Adenosine diphosphate (ADP) is one of the major platelet agonists, which are important in platelet shape changes, stability, and thrombus formation [1,2]. Platelet activation by ADP is associated with two G protein-coupled receptors (GPCRs): P2Y1, which belongs to the Gq G-proteins, and P2Y12, which is coupled with the Gi-type G-proteins. In order to activate platelets by ADP, both P2Y1 and P2Y12 receptors are necessary. However, P2Y12 is the ADP receptor that amplifies platelet responses induced by other agonists, and causes full platelets response and the secretion of platelet-dense granules [3,4].

P2Y12 has the typical structure of a 7-transmembrane G protein-coupled receptor, and plays a key role in platelet activation, the mobilization of other platelets to the side of a damaged vessel, the stabilization of aggregates, the secretion of molecules, and the enhancement of the efficiency of other agonists to activate platelets. The stimulation of P2Y12 results in the dissociation of the subunits of the heterotrimeric Gi protein coupled with the P2Y12 receptor, and the activation of the intracellular signal pathway. Since blood platelets are a very reactive type of cells, their increased activation (mainly caused by ADP) leads to complications in the circulatory system. Oral drugs based on the inhibition of the P2Y12 receptor are fundamental in pharmacotherapy in patients with acute coronary syndromes. Based on the European Society of Cardiology guidelines from 2012 to prevent secondary thrombotic episodes, patients with acute coronary syndromes (ACS) should receive dual antiplatelet therapy: an inhibitor of P2Y12 in combination with acetylsalicylic acid [5].

In recent years, our group has focused on research aimed at finding novel natural compounds with antiplatelet activity that can be used in the prevention of thrombotic episodes. We found that the bioactive components of the milk thistle (*Silybum marianum* L. Gaernt.)— flavonolignans—are very promising compounds for novel anti-platelet agents. We observed that three tested flavonolignans—silybin, silychristin, and silydianin, in a dose-dependent manner, are able to inhibit ADP-induced blood platelet aggregates and microparticles formation, as well as decrease the expression of P-selectin, and the activation of the main receptor to fibrinogen–integrin αIIbβ3. Our computer-generated models showed that all of the tested flavonolignans have conformations that are able to bind to the extracellular domain of the P2Y12 receptor, and probably block interaction with ADP. Additionally, we demonstrated that the calculated affinity of silybin and silychristin to P2Y12 is higher than the active clopidogrel metabolite [6]. For all of these reasons, we decided to verify the effect of silybin, silychristin, and silydianin on various ADP-induced platelet responses, as well as the mechanism of the P2Y12-dependent intracellular signal transduction.

## 2. Results

### 2.1. Effect of Flavonolignans on ADP Induced Blood Platelet Aggregation

**Figure 1:**
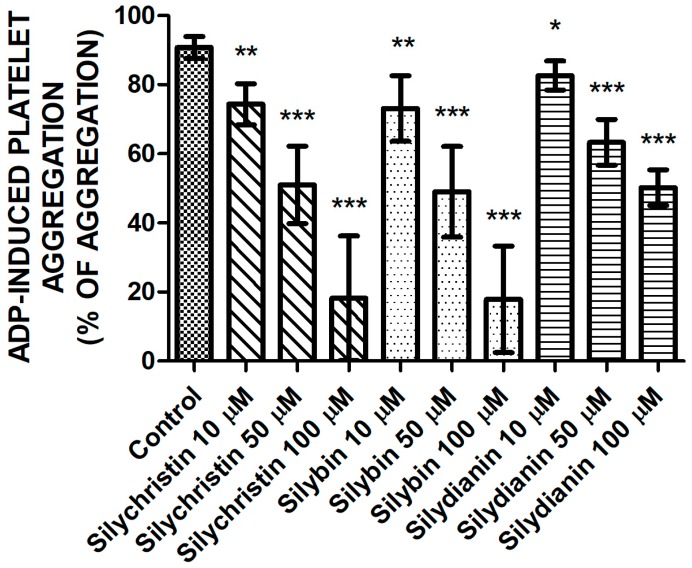
The effect of tested flavonolignans (silychristin, silybin, and silydianin) in concentrations of 10 µM, 50 µM, and 100 µM on blood platelets’ aggregation, induced by 20 µM of adenosine diphosphate (ADP) in platelet-rich plasma (PRP) samples. The data represents the mean ± SD of 16 independent experiments done in duplicate; *-*p* < 0.05, **-*p* < 0.01, ***-*p* < 0.001.

In order to verify the inhibitory effect flavonolignans on ADP-induced blood platelet activation, in the first step, we examined the effect of flavonolignans on ADP-induced blood platelets’ aggregation in platelet-rich plasma (PRP) using the light transmission aggregometry method. To ensure full platelet activation, a 20-µM ADP was added. In our measurements, we observed that in a dose-dependent manner, all of the tested compounds—silychristin, silybin, and silydianin—decrease the blood platelets’ aggregation level in PRP upon ADP stimulation (Figure 1). The strongest inhibitory effect was observed for silychristin and silybin, which at the highest used concentration (100 µM) reduced ADP-induced blood platelets’ aggregate formation (control value of 92.7%), to 18.2% and 17.8%, respectively.

### 2.2. Effect of Flavonolignans on ADP Induced Blood Platelet Adhesion to Fibrinogen

**Figure 2:**
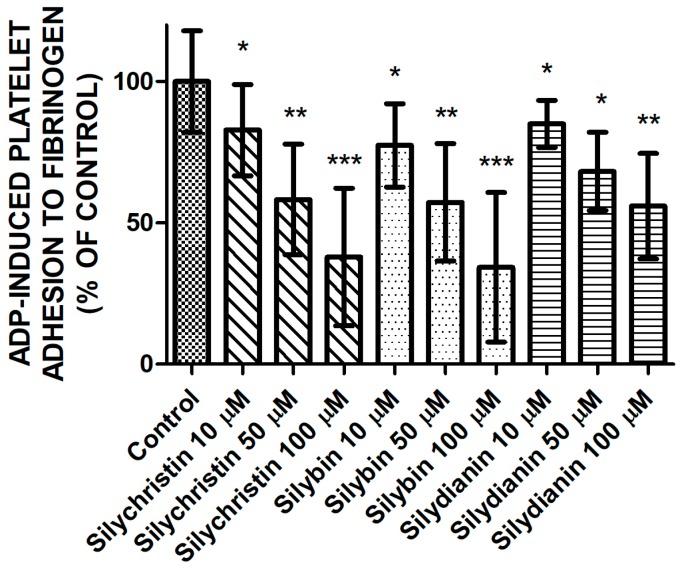
The effect of tested flavonolignans (silychristin, silybin, and silydianin) in concentrations of 10 µM, 50 µM, and 100 µM on ADP-induced blood platelets’ adhesion to fibrinogen. The data presented as a percent of control represents the mean ± SD of 16 independent experiments done in duplicate; *-*p* < 0.05, **-*p* < 0.01, ***-*p* < 0.001.

Next, we examined the effect of tested flavonolignans on the platelets’ adhesion ability induced by ADP (Figure 2). Measurements of the adhesion of isolated platelets demonstrated the reduction of ADP-induced blood platelets’ adhesion to fibrinogen in samples where platelets were treated by silychristin, silybin, and silydianin. The strongest inhibitory effect was observed for silychristin and silybin, which at the highest used concentration (100 µM) reduced ADP-induced blood platelets’ adhesion to 37.9% and 34.3% of the control, respectively.

### 2.3. Effect of Flavonolignans on PF-4 Secretion from Blood Platelets Stimulated by ADP 

**Figure 3:**
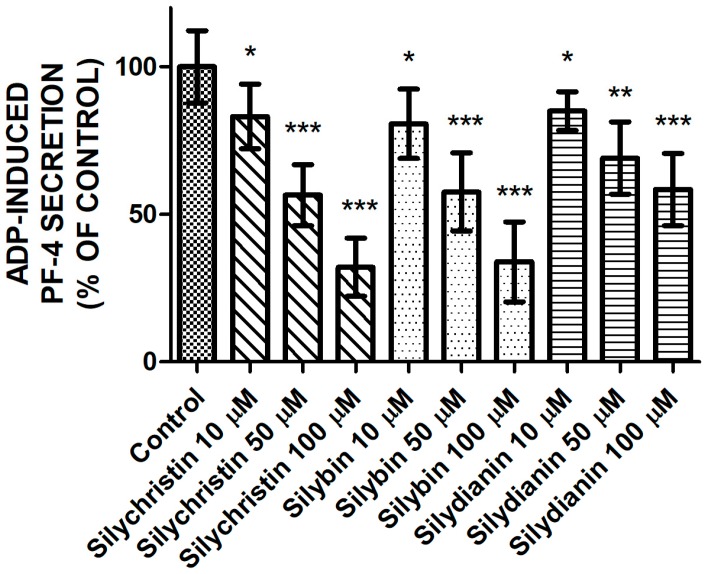
The effect of tested flavonolignans (silychristin, silybin, and silydianin) in concentrations of 10 µM, 50 µM, and 100 µM on ADP-induced PF-4 secretion from platelet α granules. The data presented as a percent of control represents the means ± SD of 16 independent experiments done in duplicate; *-*p* < 0.05, **-*p* < 0.01, ***-*p* < 0.001.

After estimating the inhibitory effect of flavonolignans on the ADP-induced aggregation and adhesion, we performed measurements of ADP-induced secretion of PF-4 from platelet α granules. Our observations also showed that flavonolignans possess a strong inhibitory effect on the blood platelet activation process by ADP. We demonstrated that the three tested flavonolignans, in all of the tested concentrations, reduced ADP-induced PF-4 secretion from blood platelets (Figure 3) to a statistically significant degree, as measured by the ELISA method.

### 2.4. Effect of Flavonolignans on Blood Platelets VASP Phosphorylation

**Figure 4:**
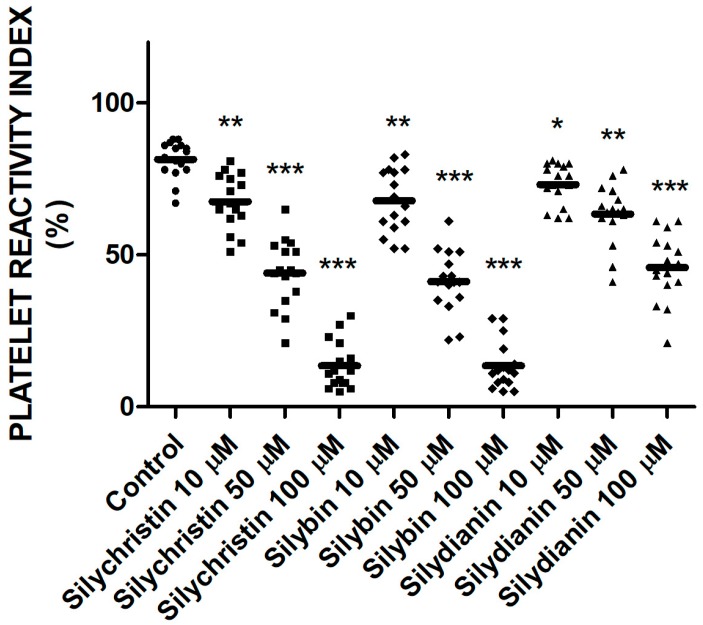
The effect of tested flavonolignans (silychristin, silybin, and silydianin) in concentrations of 10 µM, 50 µM, and 100 µM on the phosphorylation of vasodilator-stimulated phosphoprotein (VASP) protein. The data has been obtained from double-labeled flow cytometry measurements, and presented as platelet reactivity index (PRI) values. Values represent the means ± SD of 16 independent experiments; *-*p* < 0.05, **-*p* < 0.01, ***-*p* < 0.001.

To examine the effect of flavonolignans on the platelet P2Y12 receptor pathway in response to ADP, the vasodilator-stimulated phosphoprotein (VASP) assay was performed. The platelet reactivity was flow cytometry monitored in whole blood based on double labeled fluorescence. In all of the samples treated by flavonolignans, we observed that level of VASP phosphorylation was higher than in control sample (Figure 4). The highest levels of VASP phosphorylation were observed for samples treated by silychristin and silybin; at the highest used concentration (100 µM), platelet reactivity index (PRI) values were 9% and 12%, respectively versus a control value of 85%.

## 3. Discussion

The pharmacologic blockade of the P2Y12 receptor in physiologic conditions of arterial flow revealed that this receptor is essential for platelet aggregation under shear conditions. Consistent with the central role of the P2Y12 receptor in thrombosis, P2Y12 receptor antagonists reduce occlusive thrombosis in animal models. Clinical studies using clopidogrel demonstrate a significantly reduced risk of peripheral artery disease, myocardial infarction, ischemic stroke, or vascular death, in comparison with aspirin therapy. The benefits of P2Y12 antagonism have been validated by multiple clinical trials, but some of the characteristics of the present P2Y12 antagonists could be improved [3]. 

The P2Y12 receptor is an established target of antithrombotic drugs such as the thienopyridines: clopidogrel or prasugrel, or direct reversible antagonists such as ticagrelor or cangrelor. Each of these drugs has proven efficacy in large clinical trials [7]. P2Y12 antagonists are able to reduce platelet aggregation, platelet secretion, platelet–leucocyte interaction, and the contribution of platelets to the cascade of coagulation factors. Additionally, these molecules reduced thromboxane A2 (TXA2) synthesis by platelets, and thereby, also reduced the amplification of platelet functions mediated by this molecule [8]. However, the clinical practical phenomena, such as “clopidogrel resistance” due to the single nucleotide polymorphism (SNP) of cytochrome P450 or P2Y12 receptor constitutive activation, call for better antiplatelet agents. Research studies also showed that the inverse agonist of the P2Y12 receptor could play a better role over neutral antagonists. Personalized antiplatelet therapy is the most ideal destination for antiplatelet therapy in ACS patients [9].

In our previous studies, we demonstrated the strong inhibition of ADP-induced blood platelets activation by three major compounds of flavonolignans group: silybin, silychristin, and silydianin [6]. This group of compounds is present in nature in the fruits of milk thistle (*Silybum marianum* L. Gaernt.). The mixture of flavonolignans—silymarin represents 1.5–3% of the milk thistle fruit’s dry weight. The flavonolignans are very specific compounds that have different mechanism of action and bioavailability than other polyphenolic compounds. These compounds possess a unique chemical structure, which is composed of two main units. The first is based on a taxifolin, which is a flavanonol group in flavonoids. The second is a phenylpropanoid unit [10]. This structure is formed through oxidative coupling processes between flavonoids and a phenylpropanoid. The chemical structures of flavonolignans can be characterized as small, highly functionalized molecules with alternating carbocycles and heterocycles, which are very stable under Brønsted acidic conditions, while in the presences of Lewis acids or under basic conditions, the stability is reduced [11]. *Silybum marianum* has been used as a medicinal plant since the time of ancient physicians and herbalists to treat a range of liver dysfunctions and gallbladder disorders [12]. However, the studies performed in recent years demonstrated a wide range of other healthy properties of this plant, such as anticancer and anti-inflammatory actions [13]. Additionally, our research team studies confirmed the flavonolignans effect on the hemostasis system by inhibition of coagulation factors activity [14,15], as well as the reduction of the blood platelets’ response to physiological agonists such as collagen and arachidonic acid (by direct inhibition cyclooxygenase activity) [6,16,17]. However, the reduction of platelet response to collagen by flavonolignans was significantly lower than the effect on ADP-induced blood platelet activation. Additionally, we observed that these compounds are able to inhibit cross-talk between blood platelets and the immune response system [18].

In the current study, we demonstrated results that confirm our earlier observations in whole blood samples [6]. Blood platelets adhesion and aggregation are the major platelet’s functional reactions for activation. We observed a dose-dependent reduction of blood platelets’ ability to perform both ADP-induced aggregation in PRP, and the adhesion of isolated platelets to fibrinogen in samples treated with flavonolignans (Figure 1 and Figure 2). Both of these processes are dependent on conformational changes in the αIIbβ3 receptor presented in the platelets’ surface, which in its active form is responsible for platelets binding to fibrinogen. The activation of αIIbβ3 during conformational changes is related to the intracellular signalling pathway activated by the stimulation of the P2Y12 receptor [4,19], which confirms that flavonolignans interact with P2Y12 and block its activation. After comparison of the anti-adhesive and anti-aggregatory effects of the examined flavonolignans, we performed an analysis of the effect of flavonolignans on ADP-induced PF-4 secretion from platelet α granules. This experiment also showed that flavonolignans reduced the ability of blood platelets to respond to ADP (Figure 3). All of these analyses showed that silychristin and silybin have the strongest effect on blood platelet activation by ADP, while silydianin inhibits the ADP pathway, but to a lesser extent.

In order to unambiguously confirm the flavonolignans’ ability to inhibit the P2Y12 receptor, we decided to perform a flow cytometry diagnostic test dedicated to the monitoring of specific platelet P2Y12 antagonists. This assay is based on VASP phosphorylation measurements that correlate with P2Y12 receptor inhibition. VASP is a profilin and actin-binding protein, expressed in platelets at high levels (approximately 78,000 copies per platelet). VASP is crucial protein that is involved in the reorganization of the actin cytoskeleton in platelets, and the regulation of adhesive events and platelet aggregation. VASP is a main substrate of two protein kinases, PKA and PKG, which phosphorylate it at three areas: serine in positions 157 and 239, and threonine in position 278. The phosphorylation of VASP in Ser157 correlates with the reduced activation of αIIbβ3 and the inhibition of platelet aggregation, as well as a decreased ability for this protein to interact with actin [20]. VASP phosphorylation is regulated by the cAMP pathway, which is activated by Prostaglandin E1 (PGE1). Activation of the P2Y12 receptor by ADP inhibited cAMP production, which reduced VASP phosphorylation. During the assay, the VASP phosphorylation was correlated with P2Y12 receptor inhibition [21,22]. The formation and quantity of phosphorylated VASP depended on the activity of the P2Y12 receptor. A high level of the phosphorylated form of VASP can be interpreted as an effective action of the used P2Y12 antagonist [5,23]. In our flow cytometry analysis, we estimated that all of the tested flavonolignans were able to increase VASP phosphorylation in blood platelets (Figure 4). That would confirm our thesis, as well as our previous observations that these compounds are able to directly inhibit the P2Y12 receptor. Estimated PRI parameters showed that the most effective inhibitory properties possessed silybin and silychristin. Additionally, the PRI values for silybin and silychristin at the highest tested concentration (100 µM) were in the range of (5–25%), which is comparable with parameters from “good response” patients treated by P2Y12 antagonists [11,24,25].

In all of our experiments performed in this study, we used active concentrations that can be achievable in circulation during supplementation with a novel form of milk thistle extract: silymarin. Hwang et al. [26] created a novel form of silymarin—silymarin/PVP/Tween-80 at a weight ratio of 5/2.5/2.5, in which oral supplementation (equivalent to 140 mg/kg dose of silymarin) resulted in the maximum plasma concentration of silybin at level 44.85 ± 11.42 μg/mL, which corresponded to 100 μM (the maximum concentration used in this study). Additionally, the elimination half-life of unchanged forms of flavonolignans (based on silybin) is approximately 6 h [10], which is enough time for them to act as bioactive components. The used concentrations of flavonolignans were also evaluated in regard to their cytotoxicity and genotoxicity. The results demonstrated that even at a 100-µM concentration of silybin, silychristin and silydianin have neither a cytotoxic nor a genotoxic effect on blood platelets, peripheral blood mononuclear cells (PBMCs), or the human lung cancer cell lineA549. Additionally, these compounds have a protective effect on cellular mitochondria, increase the blood platelets’ mitochondrial membrane potential, and reduce the generation of reactive oxygen species in blood platelets [27].

## 4. Materials and Methods

### 4.1. Reagents

Dimethyl sulfoxide (DMSO), 3-[(3-Cholamidopropyl)dimethylammonio]-1-propanesulfonate (CHAPS), 4-(2-Hydroxyethyl)piperazine-1-ethanesulfonic acid (HEPES), glucose, heat shock bovine serum albumin, collagen type I (for adhesion), as well as the flavonolignans (silybin, silychristin and silydianin), were all obtained from the Sigma-Aldrich Chemical Co. (St. Louis, MO, USA). The Pierce BCA Protein Assay Kit came from Thermo Fisher Scientific (Waltham, MA, USA). ADP, aggregation cuvettes, and strips were purchased from Chrono-Log (Havertown, PA, USA). Frozen human plasma was obtained from whole blood collected into sodium citrate (0.32% final concentration), and was purchased from the Regional Center for Transfusion Medicine (Lodz, Poland). All of the other chemicals were reagent grade or the highest quality available.

### 4.2. Blood Samples

Blood samples were collected from 16 different healthy donors in the Centre of Laboratory Diagnostic (Lodz, Poland). All of the samples were drawn in the morning from fasting donors (minimum 12 h from last meal). Subjects did not use addictive substances and polyphenols supplementation; their diet was balanced (meat and vegetables). Each donor had a panel of diagnostic tests (morphology, C-reactive protein, alanine transaminase, aspartate transaminase, gamma-glutamyltransferase, urea, uric acid, creatinine, bilirubin, cholesterol, triglycerides, glucose, amylase, lipase, thyroid-stimulating hormone, Na^+^, K^+^, Cl^−^). We selected only donors that had no cardiovascular disorders, allergies, lipid or carbohydrate metabolism disorders, and were not being treated with any drugs. From each donor, one probe of 8.5 mL was collected (BD Vacutainer^®^ Blood Collection Tubes—ACD, Becton Dickinson, Franklin Lakes, NJ, USA). Our analysis of the blood samples was performed under the guidelines of the Helsinki Declaration for Human Research, and approved by the Committee on the Ethics of Research in Human Experimentation at the University of Lodz (Resolution No. 16/KBBN-UŁ/II/2016).

### 4.3. Isolation of Platelet-Rich Plasma and Blood Platelets

The whole blood samples were centrifuged (200 × *g*, 12 min, room temperature—RT) to isolate the PRP. The obtained PRP was then used to measure aggregation level. Blood platelets were isolated from PRP by differential centrifugation (300× *g*, 10 min, RT) to obtain a platelets pellet. The platelet-washing procedure was performed in plastic tubes, at room temperature. Washed human platelets were suspended in a modified Tyrode’s Ca^2+^/Mg^2+^ free buffer (127 mM NaCl, 2.7 mM KCl, 0.5 mM NaH_2_PO_4_, 12 mM NaHCO_3_, 5 mM HEPES, 5.6 mM glucose, pH 7.4). The platelets were counted using a photometric method according to Walkowiak et al. [28]. The final concentration of platelet suspension was 2 × 10^8^ platelets/mL.

### 4.4. Sample Preparation Protocol

The isolated platelets, PRP, as well as whole blood samples, were pre-incubated with flavonolignans (silybin, silychristin, and silydianin) in concentrations of 10 µM, 50 µM, and 100 µM, respectively, for 30 min at 37 °C. All of the tested compounds were initially dissolved in 20% DMSO to a preliminary concentration of 20 mM. Other solutions of the used compounds were also prepared in 20% DMSO. The final DMSO concentration in all of the samples was 0.1%. In the control samples, the same volume of solvent was added, and the probes were warmed for 30 min at 37 °C.

### 4.5. Blood Platelet Aggregation Induced by ADP

Platelet aggregation was measured turbidimetrically in PRP using an optical Chrono-Log aggregometer (Chrono-Log, Havertown, PA, USA). The prepared PRP samples were pre-warmed at 37 °C and stirred. After 5 min, APD (20 µM) was added to the PRP samples, and platelet aggregation was measured for 10 min. The aggregometer was calibrated each time (100% aggregation) on the platelet-poor plasma (PPP), with the appropriate concentration of each flavonolignan.

### 4.6. ADP Induced Blood Platelet Adhesion to Fibrinogen

Fibrinogen (Fg) was isolated from citrated human plasma by the cold ethanol precipitation technique according to Doolittle [29]. Fg concentration was determined spectrophotometrically at 280 nm using an extinction coefficient 1.55 for 1 mg/mL solution. The adhesion of blood platelets to Fg was determined according to Tuszynski and Murphy [30]. In the first step of the procedure, the wells of a Nunc MaxiSorp® microtiter plate (Roskilde, Denmark) were coated by Fg. The application volume of the coat protein was 100 μL per well for a 96-well plate, in a final concentration of 0.1 mg/mL. The coated microtiter plate was incubated for 16 h at 4 °C on the laboratory cradle. After incubation, to remove the unbound proteins, the microtiter plate was washed three times with 250 μL of phosphate-buffered saline (PBS). Next, to block the non-bounded places on the wells, 200 μL of 1% bovine serum albumin (heat shock) was added. The microtiter plate was incubated for 2 h at 37 °C. The excess bovine albumin was poured off, and the 96 wells were washed again three times, with 250 μL of PBS containing 0.04% Tween-20, 1 mM MgCl_2_, and 0.1 mM CaCl_2_. After that, 100 μL of platelet suspension (2 × 10^8^ platelets/mL) was added to each well, and the microtiter plate was incubated for 2 h at 37 °C. Next, the non-adherent cells were removed with three washings using 250 μL of PBS, and two washings with 250 μL of PBS containing 0.04% Tween-20, 1 mM MgCl_2_, and 0.1 mM CaCl_2_. The platelets’ adhesion level was determined by estimation of the total protein concentration using Pierce BCA Protein Assay working solutions. After 30 min incubation at 37 °C, the microtiter plate was measured with spectrophotometry at 562 nm using a BMG LABTECH SPECTROstar Nano platelet reader (Ortenberg, Germany).

### 4.7. Platelet Factor 4 Secretion

The estimation of PF-4 secretion by blood platelets was performed using a commercial human PF-4 ELISA kit RayBiotech (Norcross, GA, USA). The secretion of PF-4 by blood platelets was monitored in the PRP, after stimulation with ADP (20 µM). PRP samples (control and pre-incubated with the flavonolignans) were activated by ADP (10 min, 37 °C), and then immediately centrifuged in order to remove the blood platelets and obtain PPP. Before the PF-4 immunodetection, the PPP samples were diluted 200 times with PBS. All of the steps of the analysis were performed according to the manufacturer’s protocol. The measurement was performed using the BMG LABTECH SPECTROstar Nano platelet reader (Ortenberg, Germany).

### 4.8. Estimation of Platelet Vasodilator-Stimulated Phosphoprotein (VASP) Phosphorylation

The response of blood platelets to ADP with the phosphorylation of focal adhesion vasodilator-stimulated phosphoprotein (VASP) in platelets was also investigated using the flow cytometrical VASP assay (PLTVASP/P2Y12) from BioCytex (Marseille, France). The whole procedure was performed on prepared whole blood samples according to the manufacturer’s protocol. Analysis was performed using a CUBE 6 flow cytometer with CyView Software v. 1.5.5.8 - PARTEC (Münster, Germany). Platelet response was expressed as platelet reactivity index (PRI) values.

### 4.9. Statistical Analysis

The calculated figures were statistically analyzed using the program StatsDirect® Version 2.7.2 (Cheshire, UK). First, the Shapiro–Wilk test was used to assess the normal distribution of the variables. Next, the results were analyzed for equality of variance using Levene’s test. The significance of the differences between the values was analyzed using analysis of variance, followed by Tukey’s range test for multiple comparisons (for data with normal distribution and equality of variance), or the Kruskal-Wallis test for non-parametric comparisons (when variables had other than normal distribution or had no equality of variance); *p* < 0.05 was accepted as statistically significant [31,32,33,34,35].

## 5. Conclusions

The results obtained in this study clearly demonstrated that three major flavonolignans, silybin, silychristin, and silydianin, have inhibitory properties against the P2Y12 receptor, and block ADP-induced blood platelet activation. This effect might encourage the use of flavonolignans as nutritional supplements, as well as future medicinal agents in treatment against thrombotic events where the hyperreactivity of blood platelets is involved. Additionally, flavonolignans’ molecular structures could be also used as pharmacophores to design and synthesize novel P2Y12 antagonists.

## References

[1] Daniel J.L., Dangelmaier C., Jin J., Ashby B., Smith J.B., Kunapuli S.P. (1998). Molecular basis for adp-induced platelet activation. I. Evidence for three distinct adp receptors on human platelets. J. Biol. Chem..

[2] Savi P., Beauverger P., Labouret C., Delfaud M., Salel V., Kaghad M., Herbert J.M. (1998). Role of p2y1 purinoceptor in adp-induced platelet activation. FEBS Lett..

[3] Dorsam R.T., Kunapuli S.P. (2004). Central role of the p2y12 receptor in platelet activation. J. Clin. Invest..

[4] Kahner B.N., Shankar H., Murugappan S., Prasad G.L., Kunapuli S.P. (2006). Nucleotide receptor signaling in platelets. J. Thromb. Haemost..

[5] Wiviott S.D., Braunwald E., McCabe C.H., Montalescot G., Ruzyllo W., Gottlieb S., Neumann F.J., Ardissino D., De Servi S., Murphy S.A. (2007). Prasugrel versus clopidogrel in patients with acute coronary syndromes. N. Engl. J. Med..

[6] Bijak M., Szelenberger R., Saluk J., Nowak P. (2017). Flavonolignans inhibit adp induced blood platelets activation and aggregation in whole blood. Int. J. Biol. Macromol..

[7] Gachet C., Hechler B. (2013). The p2y receptors and thrombosis. WIREs Membr. Transp. Signal.

[8] Kim S., Kunapuli S.P. (2011). P2y12 receptor in platelet activation. Platelets.

[9] Zhang Y., Zhang S., Ding Z. (2017). Role of p2y_12_ receptor in thrombosis. Adv. Exp. Med. Biol..

[10] Bijak M. (2017). Silybin, a major bioactive component of milk thistle (silybum marianum l. Gaernt.)-chemistry, bioavailability, and metabolism. Molecules.

[11] Aleil B., Jacquemin L., De Poli F., Zaehringer M., Collet J.P., Montalescot G., Cazenave J.P., Dickele M.C., Monassier J.P., Gachet C. (2008). Clopidogrel 150 mg/day to overcome low responsiveness in patients undergoing elective percutaneous coronary intervention: Results from the vasp-02 (vasodilator-stimulated phosphoprotein-02) randomized study. JACC Cardiovasc. Interv..

[12] Abenavoli L., Capasso R., Milic N., Capasso F. (2010). Milk thistle in liver diseases: Past, present, future. Phytother. Res..

[13] Bijak M. (2017). [flavonolignans - compounds not only for liver treatment]. Pol. Merkur. Lekarski.

[14] Bijak M., Ponczek M.B., Nowak P. (2014). Polyphenol compounds belonging to flavonoids inhibit activity of coagulation factor x. Int. J. Biol. Macromol..

[15] Bijak M., Ziewiecki R., Saluk J., Ponczek M., Pawlaczyk I., Krotkiewski H., Wachowicz B., Nowak P. (2014). Thrombin inhibitory activity of some polyphenolic compounds. Med. Chem. Res..

[16] Bijak M., Saluk-Bijak J. (2017). Flavonolignans inhibit the arachidonic acid pathway in blood platelets. BMC Complement. Altern. Med..

[17] Bijak M., Dziedzic A., Saluk-Bijak J. (2018). Flavonolignans reduce the response of blood platelet to collagen. Int. J. Biol. Macromol..

[18] Bijak M., Dziedzic A., Synowiec E., Sliwinski T., Saluk-Bijak J. (2017). Flavonolignans inhibit il1-β-induced cross-talk between blood platelets and leukocytes. Nutrients.

[19] Offermanns S. (2006). Activation of platelet function through g protein-coupled receptors. Circ. Res..

[20] Wentworth J.K., Pula G., Poole A.W. (2006). Vasodilator-stimulated phosphoprotein (vasp) is phosphorylated on ser157 by protein kinase c-dependent and -independent mechanisms in thrombin-stimulated human platelets. Biochem. J..

[21] Gurbel P.A., Bliden K.P., Hiatt B.L., O’Connor C.M. (2003). Clopidogrel for coronary stenting: Response variability, drug resistance, and the effect of pretreatment platelet reactivity. Circulation.

[22] Müller I., Besta F., Schulz C., Massberg S., Schönig A., Gawaz M. (2003). Prevalence of clopidogrel non-responders among patients with stable angina pectoris scheduled for elective coronary stent placement. Thromb. Haemost..

[23] Bagoly Z., Sarkady F., Magyar T., Kappelmayer J., Pongrácz E., Csiba L., Muszbek L. (2013). Comparison of a new p2y12 receptor specific platelet aggregation test with other laboratory methods in stroke patients on clopidogrel monotherapy. PLoS ONE.

[24] Pampuch A., Cerletti C., de Gaetano G. (2006). Comparison of vasp-phosphorylation assay to light-transmission aggregometry in assessing inhibition of the platelet adp p2y12 receptor. Thromb. Haemost..

[25] Gorog D.A., Fuster V. (2013). Platelet function tests in clinical cardiology: Unfulfilled expectations. J. Am. Coll. Cardiol..

[26] Hwang d.H., Kim Y.I., Cho K.H., Poudel B.K., Choi J.Y., Kim D.W., Shin Y.J., Bae O.N., Yousaf A.M., Yong C.S. (2014). A novel solid dispersion system for natural product-loaded medicine: Silymarin-loaded solid dispersion with enhanced oral bioavailability and hepatoprotective activity. J. Microencapsul..

[27] Bijak M., Synowiec E., Sitarek P., Sliwiński T., Saluk-Bijak J. (2017). Evaluation of the cytotoxicity and genotoxicity of flavonolignans in different cellular models. Nutrients.

[28] Walkowiak B., Michalak E., Koziolkiewicz W., Cierniewski C.S. (1989). Rapid photometric method for estimation of platelet count in blood plasma or platelet suspension. Thromb. Res..

[29] Doolittle R.F., Schubert D., Schwartz S.A. (1967). Amino acid sequence studies on artiodactyl fibrinopeptides. I. Dromedary camel, mule deer, and cape buffalo. Arch. Biochem. Biophys..

[30] Tuszynski G.P., Murphy A. (1990). Spectrophotometric quantitation of anchorage-dependent cell numbers using the bicinchoninic acid protein assay reagent. Anal. Biochem..

[31] Bijak M., Kolodziejczyk-Czepas J., Ponczek M.B., Saluk J., Nowak P. (2012). Protective effects of grape seed extract against oxidative and nitrative damage of plasma proteins. Int. J. Biol. Macromol..

[32] Bijak M., Nowak P., Borowiecka M., Ponczek M.B., Zbikowska H.M., Wachowicz B. (2012). Protective effects of (-)-epicatechin against nitrative modifications of fibrinogen. Thromb. Res..

[33] Bijak M., Saluk J., Antosik A., Ponczek M.B., Zbikowska H.M., Borowiecka M., Nowak P. (2013). Aronia melanocarpa as a protector against nitration of fibrinogen. Int. J. Biol. Macromol..

[34] Bijak M., Saluk J., Tsirigotis-Maniecka M., Komorowska H., Wachowicz B., Zaczynska E., Czarny A., Czechowski F., Nowak P., Pawlaczyk I. (2013). The influence of conjugates isolated from matricaria chamomilla l. On platelets activity and cytotoxicity. Int. J. Biol. Macromol..

[35] Zbikowska H.M., Antosik A., Szejk M., Bijak M., Olejnik A.K., Saluk J., Nowak P. (2014). Does quercetin protect human red blood cell membranes against γ-irradiation?. Redox Rep..

